# Real-world experience with rituximab for multiple sclerosis treatment in a Brazilian tertiary center

**DOI:** 10.1055/s-0046-1820528

**Published:** 2026-05-18

**Authors:** Ana Beatriz Simon-Nogueira, Arthur Cesário de Holanda, Rafael Augusto Rosalem, Thiago Ivan Vilchez Santillan, Guilherme Diogo Silva, Samira Luisa Apostolos-Pereira, Tarso Adoni, Dagoberto Callegaro, Mariana Gondim Spricigo, Mateus Boaventura de Oliveira

**Affiliations:** 1 Universidade de São Paulo, Faculdade de Medicina, Hospital das Clínicas, Divisão de Neurologia, Grupo de Neuroimunologia, São Paulo SP, Brazil.

**Keywords:** Multiple Sclerosis, Rituximab, Effectiveness, Safety

## Abstract

**Background:**

Rituximab, an anti-cluster of differentiation 20 (anti-CD20) monoclonal antibody, has been widely used off-label for multiple sclerosis (MS) treatment, demonstrating high effectiveness and a favorable safety profile. However, real-world data from Brazil, particularly within the public healthcare system, remain scarce.

**Objective:**

To assess the real-world effectiveness and safety of rituximab for MS treatment in a Brazilian tertiary center.

**Methods:**

We conducted a single-center, observational study at Hospital das Clínicas da Faculdade de Medicina da Universidade de São Paulo (HCFMUSP), including MS patients treated with rituximab between January 2016 and October 2024. The primary outcome was the change in annualized relapse rate (ARR) following rituximab initiation. The secondary outcomes included disability progression, radiological activity, and safety. The statistical analyses employed non-parametric tests and time-to-event analysis.

**Results:**

Among the 45 patients (25 with relapsing-remitting MS [RRMS], 18 with secondary progressive MS, and 2 with primary progressive MS), rituximab reduced the ARR by 89.8% (from 0.49–0.05; *p* < 0.001), with 90.9% remaining relapse-free. In RRMS patients, the ARR significantly decreased, from 0.56 to 0.17, after rituximab initiation ( *p* = 0.019), with 88% remaining relapse-free (95%CI: 68.8–97.5). Radiological activity was observed in 6.8% of the sample. Patients with breakthrough disease activity (n = 4; 8.9%) presented more frequent prior natalizumab use than those without it (75.0% versus 43.9%; *p* = 0.039), as well as higher pretreatment relapse rates (1.8 versus 0.7; *p* = 0.033). Infusion-related reactions affected 12 (26.7%) patients, and they were mostly mild, while infections were the most common non-infusion adverse event (20; 57.8%). In total, 4 patients discontinued rituximab, mainly due to access limitations.

**Conclusion:**

Rituximab demonstrated high effectiveness and acceptable safety for MS treatment in a Brazilian public healthcare setting. Its favorable risk-benefit profile supports broader adoption in resource-limited contexts.

## INTRODUCTION

Multiple sclerosis (MS) is an inflammatory demyelinating disease that affects the central nervous system (CNS), triggered by environmental factors in genetically-susceptible individuals. Initially, transient myelin damage with remyelination leads to relapsing-remitting MS (RRMS), 1 but chronic microglial activation and neurodegeneration eventually cause progressive disability. Untreated patients typically transition to secondary progressive MS (SPMS) within 20 years 2 A subset presents with primary progressive MS (PPMS) since onset. 3 Worldwide, MS affects about 2.8 million people, mainly young women, and is the leading non-traumatic cause of disability in young adults. 4

Although traditionally accepted as a T-cell mediated disease, the participation of B-cells has gained recognition 5 in both systemic and CNS-compartmentalized inflammatory responses. Additionally, B-cell depleting therapies have been shown to be effective in reducing inflammatory activity in MS. Monoclonal antibodies targeting specific fragment antigen-binding (Fab) domains of cluster of differentiation 20-positive (CD-20 + ) lymphocytes have been investigated for MS treatment for nearly 2 decades. 6 The trials called A Study to Evaluate Rituximab in Adults with Relapsing Remitting Multiple Sclerosis (HERMES) and A Study to Evaluate the Safety and Efficacy of Rituximab in Adults with Primary Progressive Multiple Sclerosis (OLYMPUS) were the first to assess the efficacy and safety of rituximab for RRMS and PPMS with positive results, especially in patients with high inflammatory burden. 7 8 Although rituximab has not been approved for the on-label treatment of MS, other therapies have emerged as effective alternatives and are now approved for on-label use. Ocrelizumab, for instance, demonstrated high efficacy and safety in pivotal clinical trials such as A Study of Ocrelizumab in Comparison with Interferon Beta-1a (Rebif) in Participants with Relapsing Multiple Sclerosis (OPERA), 9 Study to Evaluate the Effectiveness and Safety of Ocrelizumab in Participants with Early Stage Relapsing Remitting Multiple Sclerosis (RRMS) (ENSEMBLE), 10 and A Study of Ocrelizumab in Participants with Primary Progressive Multiple Sclerosis (ORATORIO). 11 Similarly, ofatumumab gained approval based on the Efficacy and Safety of Ofatumumab Compared to Teriflunomide in Patients with Relapsing Multiple Sclerosis (ASCLEPIOS) studies, 12 and ublituximab was supported by the Study to Assess the Efficacy and Safety of Ublituximab in Participants with Relapsing Forms of Multiple Sclerosis (RMS) (ULTIMATE) trials. 13

Over the years, although rituximab is still considered an off-label treatment, randomized trials and observational studies have consistently demonstrated its high efficacy and favorable safety profile, particularly for RRMS. 14 15 16 The Comparison between All Immunotherapies for Multiple Sclerosis (COMBAT-MS) study, 17 for instance, compared the efficacy and safety outcomes of low-dose rituximab with other disease-modifying therapies (DMTs) approved for MS in Sweden. The results showed an annualized relapse rate (ARR) of 0.03 over 3 years, with 75.7% to 82.1% of the patients achieving no evidence of disease activity (NEDA-3) status, including absence of relapses, radiological activity, and confirmed disability worsening. In the same study, 8 rituximab demonstrated efficacy similar to that of natalizumab and was superior to the other DMTs evaluated. The Rituximab versus Fumarate in Newly Diagnosed Multiple Sclerosis (RIFUND-MS) trial, 14 a multicenter, rater-blinded, phase-3 randomized controlled study conducted at 17 Swedish hospitals, demonstrated a relative risk reduction of 81% in protocol-defined relapses with rituximab compared to dimethyl fumarate in MS patients.

A meta-analysis including 20 studies on the safety and efficacy of rituximab 18 reported an average absolute reduction in the ARR of 1.00 for RRMS patients, and a relapse-free rate of 86.2% after 96 weeks. Additionally, there was a mean reduction of 0.62 in the Expanded Disability Status Scale (EDSS) score. Infusion-related events (IREs) were observed in 31% of the patients, while infections were reported in 33%. 18 However, most available data come from European and North American cohorts, with limited evidence from the Brazilian population. This is particularly relevant given rituximab's favorable cost-effectiveness, 19 considering middle- to low-income settings like Brazil, where most patients rely on the public healthcare system.

The current study aims to describe the real-world experience of a Brazilian tertiary center with rituximab for MS treatment, evaluating its effectiveness and safety, and to generate local data that may support its broader incorporation into the public health system.

## METHODS

### Study design

We conducted a single-center observational study with retrospective and prospective data of people with MS (pwMS) under regular follow-up at the outpatient clinic of Hospital das Clínicas da Faculdade de Medicina da Universidade de São Paulo (HCFMUSP). Data was collected from March 1st, 2024, to December 11th, 2024, and the study included patients who received rituximab between January, 2016, and October, 2024. All patients were initially identified retrospectively up to June 1, 2024, and subsequently followed prospectively with continued clinical and radiological assessments until December 11, 2024. Clinical data, including magnetic resonance imaging (MRI) findings, laboratory results, and disease activity, were collected up to the predefined analysis cutoff date. The study was approved by the institutional Ethics Committee (under no. 61659422.4.0000.0068), and all participants provided written informed consent prior to their inclusion.

### Patient selection

Rituximab is not available for MS treatment in the Brazilian public healthcare system. However, at our center, it can be prescribed to a limited number of patients. The main eligibility criteria include current or previous natalizumab treatment with progressive multifocal leukoencephalopathy (PML) risk in patients whose treatment with other available drugs has failed, and in selected cases of progressive disease. Since natalizumab is largely available in our public health system, naïve treatment with rituximab is extremely uncommon.

Patients with current or previous use of rituximab were identified during the outpatient clinic routine. Their medical records were then reviewed for eligibility using the following inclusion criteria: minimum age of 18 years at data collection; diagnosis of MS according to the 2017 revisions of the McDonald criteria, 20 regardless of phenotype; and exposure to rituximab and clinical follow-up of at least 6 months after first infusion. Those without documented clinical evaluation after rituximab infusion or with insufficient information were excluded from analysis.

### Data collection and study outcomes

Data was collected regarding demographic variables (sex, age, ethnicity), clinical characteristics (MS type, disease duration, disease activity, EDSS scores before and after the MS treatment, radiological activity at baseline and during the follow-up, and adverse events due to rituximab as available on the medical records at the last follow-up recorded.

The primary outcome was the assessment of the AAR during rituximab treatment. Relapse was defined as the subacute onset of new or worsening neurological symptoms consistent with MS, lasting more than 24 hours, and preceded by at least 30 days of clinical stability. 20 The secondary efficacy outcomes included evaluating disease progression through the EDSS score every 6 months, as well as the presence of radiological activity during follow-up, defined as new/enlarging lesions on T2-weighted sequences or gadolinium-enhancement on MRI scans. Confirmed EDSS progression was considered as an increase in the EDSS score by 1 point if the baseline score was ≤ 5.5, or a 0.5-point increase if the baseline score was > 5.5. 21 Safety was assessed based on the incidence of adverse events, categorized as IREs and non-IREs. Additionally, we analyzed the rates of drug discontinuation and the reasons for discontinuation.

### Statistical analysis

Statistical analyses were performed using IBM SPSS Statistics for Windows (IBM Corp.) software, version 20.0. Group comparisons were made using the Mann-Whitney, Chi-squared or Fisher's exact tests, depending on the type of variable. Values of *p* < 0.05 were considered statistically significant. The time until rituximab discontinuation was presented through a Kaplan-Meier curve.

## RESULTS

**Figure 1:**
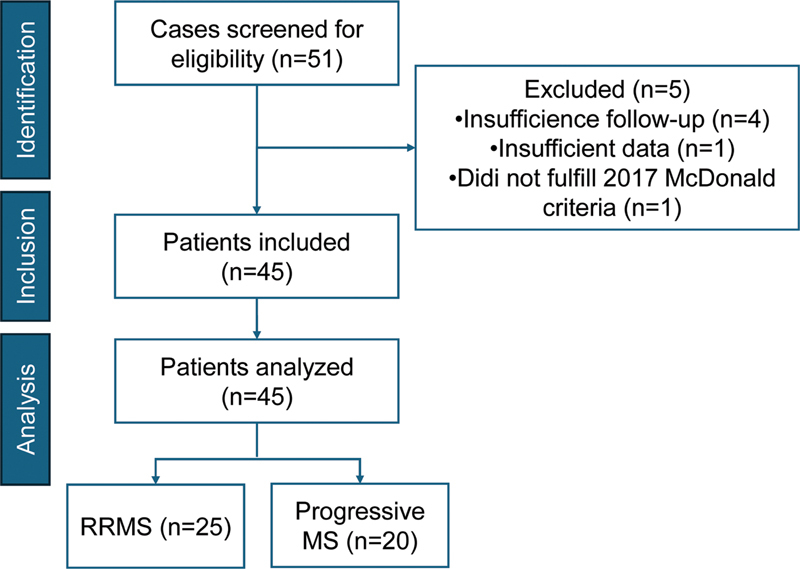
Flow chart of patient selection.

The present study included 45 pwMS: 25 with RRMS, 18 with SPMS, and 2 with PPMS. The patient selection process is illustrated in Figure 1 . Rituximab infusions were administered between January 7, 2016, and December 4, 2024. The demographic and clinical characteristics of the cohort are summarized in Table 1 .

**Table 1:** Demographic and clinical characteristics of patients at baseline and follow-up

		Total (n = 45)	RRMS (n = 25)	Progressive MS (n = 20)
Female sex: n (%)		31 (68.9)	17 (68)	14 (70)
White race: n (%)		21 (46.7)	13 (52)	8 (40)
Age at onset (years): median (IQR)		28 (22.7–33.8)	26.2 (21.2–30.3)	33.6 (24.3–40.4)
Age at rituximab initiation (years): median (IQR)		40.9 (35–48.1)	37.5 (30.7–42.1)	46.1 (40.8–56)
Disease duration (years): median (IQR)		13.8 (9.8–17.9)	12.6 (9.5–16.8)	15.1 (11.9–20)
Mean number of relapses in the 12 months prior to rituximab		0.5 ± 0.7	0.7 ± 0.8	0.3 ± 0.6
Mena number of relapses in the 24 months prior to rituximab		0.8 ± 1.1	1.1 ± 1.3	0.4 ± 0.6
**Patients with relapses in the 24 months prior to rituximab: n (%)**		**21 (46.7)**	**15 (60)**	**6 (30)**
EDSS score at baseline: median (IQR)		6.0 (3.5–6.5)	4.0 (2.5–6.0)	6.5 (6.5–7.5)
Presence of GELs on baseline MRI: n (%)		6 (13.3)	5 (20)	1 (5)
Number of previous DMTs: median (IQR)		4 (3–5.5)	4 (3–5.5)	4.5 (3.5–6)
Prior treatment: n (%)	* Natalizumab*	21 (46.7)	9 (36)	12 (60)
	* Fingolimod*	17 (37.8)	12 (48)	5 (25)
	* Dimethyl fumarate*	3 (6.7)	1 (4)	2 (10)
	* Cladribine*	1 (2.2)	1 (4)	0
	* Ocrelizumab*	1 (2.2)	1 (4)	0
	* Cyclophosphamide*	1(2.2)	1 (4)	0
	* Naïve*	1 (2.2)	−	1 (5%)
JCV + serology: n (%)		38 (84.4)	22 (88)	16 (80)
Washout period (days): median (IQR)		36 (25.2–68.4)	36 (25.2– 50.4)	46.8 (22.8–291.6)
2,000 mg in the first treatment course: n (%)		38 (84.4)	20 (80)	18 (90)
Duration of rituximab treatment (months): median (IQR)		21.4 (12.6– 32.3)	25.5 (12.6–36.3)	20.2 (12.7– 25.8)
Number of treatment courses: median (IQR)		3 (2–4)	4 (2–6)	3 (2–4)

Abbreviations: DMTs, disease-modifying therapies; EDSS, Expanded Disability Status Scale; GELs, gadolinium-enhancing lesions; IQR, interquartile range; JCV, John Cunningham virus; MRI, magnetic resonance imaging; MS, multiple sclerosis; RRMS, relapsing-remitting multiple sclerosis.

Most patients were white women, with a median age of 40.9 (interquartile range [IQR]: 35–48.1) years at the initiation of rituximab. The median disease duration at baseline was of 13.8 (IQR: 9.8–17.9) years. Among the RRMS patients, the most common DMT prior to rituximab was fingolimod (12/25; 48%), whereas natalizumab was the most common for those with progressive phenotypes (12/20; 60%). Of the 21 pwMS who switched directly from natalizumab to rituximab, the main reason was the risk of PML (13/61.9%), while in 2/9.5% of cases, the reason was inflammatory activity under natalizumab treatment. Nearly all patients transitioning from fingolimod (16/17; 94.2%) were already contraindicated for natalizumab treatment, due to presence of John Cunningham virus (JCV) antibodies and more than 24 infusions. Only 1 patient initiated rituximab as a first DMT, which was prescribed through private health insurance.

The median duration of the rituximab treatment was of 21.4 months, with the number of infusions per patient ranging from 1 to 16. The primary reasons for switching to rituximab were therapeutic failure of the previous DMT (22/48.9%), followed by the risk of PML (14/31.1%). Adverse events and poor adherence to prior DMTs accounted for 6.7% each.

### Primary outcome

**Figure 2:**
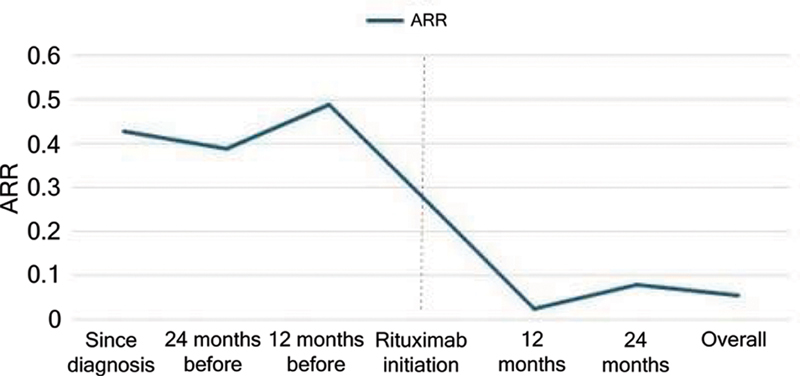
Annualized relapse rate (ARR) throughout time, before and after rituximab treatment.

The overall ARR decreased from 0.49 before rituximab to 0.05 after its initiation ( *p* < 0.001), corresponding to an 89.8% relative reduction ( Figure 2 ). During the follow-up period, 90.9% (95 CI: 82.5–99.3) of the patients remained relapse-free. Comparisons between patients with and without clinical relapse are detailed in Table 2 . Among the 25 RRMS patients, ARR decreased from 0.56 to 0.17 after rituximab ( *p* = 0.019), with 88% remaining relapse-free (95%CI: 68.8–97.5). Prior use of natalizumab immediately before rituximab (43.9% versus 75%; *p* = 0.039), along with a higher relapse frequency in the 24 months (mean number of relapses of 0.7 in the group without clinical relapses vs 1.8 in the group with clinical relapses after rituximab; *p* = 0.033) leading up to rituximab initiation, were associated with an increased risk of relapse.

**Table 2:** Comparative analysis of patients on rituximab with and without clinical relapse

		Without clinical relapse (n = 41)	Clinical relapse (n = 4)	*p* -value
Female sex: n (%)		28 (68.2)	3 (75)	0.634
RRMS phenotype: n (%)		22 (53.7)	3 (75)	0.690
Mean age at baseline (years)		41.7 ± 11.0	44.9 ± 7.1	0.452
Mean number of relapses prior to rituximab		6.0 ± 3.3	8.5 ± 1.3	0.091
Mean number of relapses in the last 12 months prior to rituximab		0.5 ± 0.7	0.8 ± 1.0	0.577
Mean number of relapses in the last 24 months prior to rituximab		0.7 ± 1.1	1.8 ± 1.0	0.033*
Presence of GELs on baseline MRI: n (%)		5 (19.2; n = 26)	1 (25)	0.676
Washout period (days): median (IQR)		36 (25.2–68.4)	39.6 (28.8–54)	0.686
Previous DMT: n (%)	Natalizumab	18 (43.9)	3 (75%)	0.039*
	Fingolimod	17 (41.6)	0	
	Cyclophosphamide	0	1 (25%)	
	Other	6 (14.6)	0	
Number of rituximab courses: median (IQR)		3 (2–4)	5.5 (3.5–7.0)	0.250
Duration of rituximab treatment (months): median (IQR)		20.9 (12.6–41.2)	34.2 (21.5–47.3)	0.189

Abbreviations: DMT, disease-modifying therapy; GELs, gadolinium-enhancing lesions; IQR, interquartile range; MRI, magnetic resonance imaging; RRMS, relapsing-remitting multiple sclerosis.

Note: The Mann-Whitney U and Chi-squared tests were perfomed as appropriate.

Five events compatible with relapse were reported among 4 patients, 2 of which were associated with concomitant radiological activity. The median age of these patients was of 43.5 (range: 42–55) years. Natalizumab was the most common preceding DMT (3/4; 75%), with a median washout period of 39.6 (range: 20–50) days. The median duration of rituximab use was of 34.5 months, and only 1 patient experienced a significant delay of more than 3 months on average between doses, due to difficulties in accessing the medication. The demographic and clinical characteristics of each patient who experienced relapse after rituximab initiation are described in **Supplementary Material Table S1** (available at: https://www.arquivosdeneuropsiquiatria.org/wp-content/uploads/2026/01/ANP-2025.0376-Supplementary-Material-I.docx ).

### Secondary efficacy outcomes

**Figure 3:**
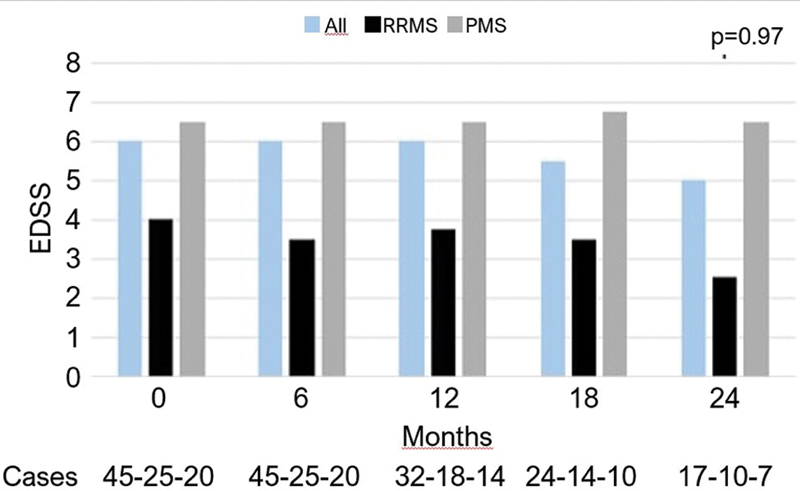
Expanded Disability Status Scale (EDSS) throughout time, and according to disease subtype, in patients treated with rituximab.

Confirmed disability worsening, assessed through the EDSS score, was observed in 13.3% of the patients, primarily among those with progressive phenotypes compared to RRMS patients (20% versus 8%). No significant change in EDSS scores was observed over a 24-month period in the overall population (6.0 versus 5.0 respectively; *p* = 0.97) ( Figure 3 ). **Supplementary Material Table S2** (available at https://www.arquivosdeneuropsiquiatria.org/wp-content/uploads/2026/01/ANP-2025.0376-Supplementary-Material-II.docx ) provides detailed pre- and posttreatment data.

Radiological activity was observed in 3 patients, corresponding to 6.8% of the overall cohort; however, MRI follow-up was only available for 19 patients, among whom radiological activity was detected in 15.8%. Limited access to MRI in our public health system, particularly for patients with less than 1 year of rituximab exposure, accounted for incomplete follow-up imaging. NEDA-3 was achieved by 77.8% of the patients (95%CI: 65.7–89.9).

### Safety outcomes

Infusion-related events were reported in 26.7% of the patients, most of which were mild (grade 1 or 2; Table 3 ). A total of 4 patients (8.3%) experienced grade-4 IREs, but none required hospitalization or treatment discontinuation.

**Table 3:** Infusion-related events with rituximab therapy

Infusion related events	12 (26.7%)
Grade 1A (n = 4)	4 (8.9%)
Grade 1B (n = 1)	1 (2.2%)
Grade 2 (n = 4)	4 (8.9%)
Grade 3 (n = 2)	2 (4.4%)
Grade 4 (n = 1)	1 (2.2%)

Non-IREs were reported by 57.8% of the patients during follow-up. The most common adverse events were infections and laboratory abnormalities ( Table 4 ). Upper respiratory and urinary tract infections were the most frequent, with 50% requiring antibiotics and 15% (3 patients) leading to hospitalization. Hospitalized patients predominantly had higher EDSS scores (all 3 patients had EDSS scores ≥ 6.5).

**Table 4:** Non-infusion-related events with rituximab therapy

Reported non-infusion-related events (n = 49)	
Infection Urinary tract Upper respiratory tract Pneumonia Infectious diarrhea Herpetic infection Other	20 (40.8%) 9 (18.4%) 6 (12.2%) 1 (2.0%) 2 (4.1%) 1 (2.0%) 1 (2.0%)
Hospitalization	3 (6.1%)
Laboratory abnormalities Hypogammaglobulinemia Lymphopenia Neutropenia	21 (40.8%) 16 (32.7%) 4 (8.2%) 1 (4.1%)
Skin reactions	2 (4.1%)
Fatigue	1 (2.1%)
Headache	1 (2.1%)
Wearing-off symptoms	1 (2.1%)

Hypogammaglobulinemia (defined as levels of immunoglobulin G [IgG] < 565 mg/dL or of immunoglobulin M [IgM] < 40 mg/dL, according to threshold values used by Houser et al. 22 ) was the most common laboratory abnormality, affecting 35.6% (16/45) of the patients. When considering only IgG levels, 17.8% (8/45) were affected; however, none required immunoglobulin replacement therapy. Lymphopenia was observed in 8.9% (4/45), and mild neutropenia occurred in 2.2% (1/45), all of which resolved spontaneously without infectious complications. No cases of febrile neutropenia occurred in our cohort.

**Fig. 4:**
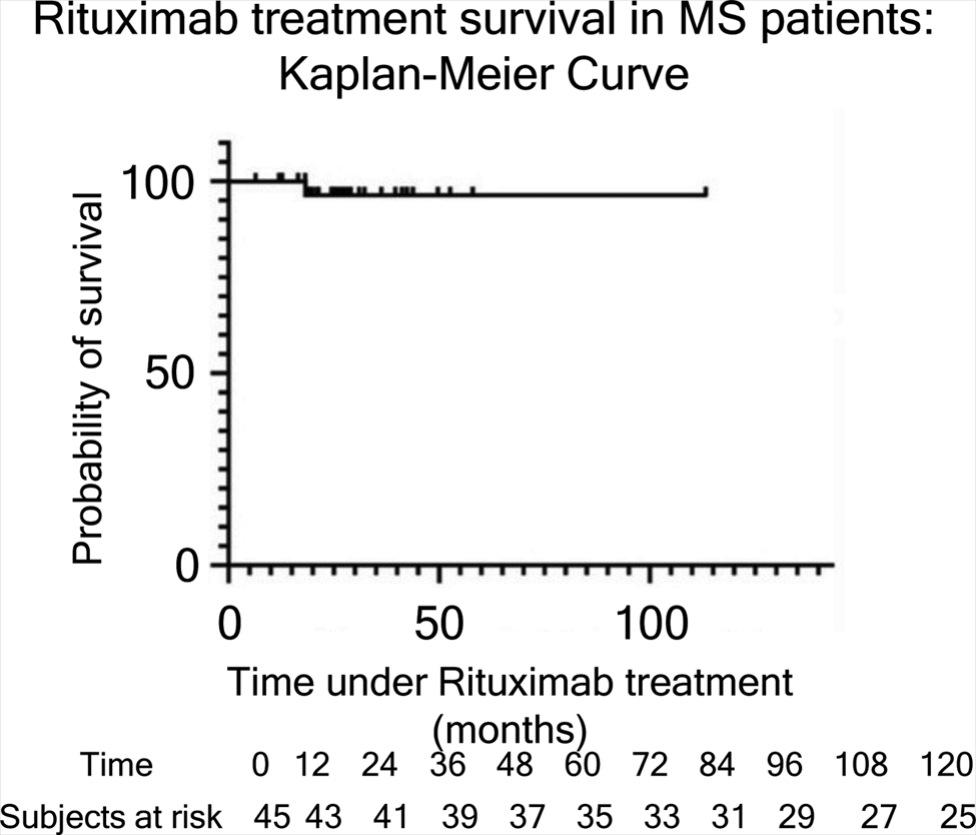
Kaplan-Meier survival analysis of rituximab, adjusted for treatment accessibility.

A total of 4 patients (8.9%) discontinued rituximab during the study period. The primary reason for discontinuation was difficulty accessing the medication (3/4; 75%), as rituximab is not readily available for MS treatment in the Brazilian public health system. Only 1 patient discontinued rituximab due to lack of efficacy and subsequently transitioned to alemtuzumab. Drug survival, excluding administrative discontinuations, is illustrated in the Kaplan-Meier curve shown in Figure 4 .

## DISCUSSION

The current study provides further evidence supporting the effectiveness and safety of rituximab in pwMS, particularly in Brazil. Following rituximab initiation, we observed a substantial reduction in disease activity, with an overall AAR of 0.05. Additionally, 90.9% of the patients remained relapse-free, and 77.8% achieved NEDA-3 status. The most common adverse events included infections, primarily mild urinary tract and respiratory infections, as well as laboratory abnormalities such as hypogammaglobulinemia. Neither IREs nor non-iIREs led to treatment discontinuation in our cohort. Instead, the primary reason for discontinuation was limited access to rituximab in Brazil, highlighting the significant barriers to treatment availability.

The AAR found in the present study is similar to the rates reported in other observational studies with rituximab (0.03–0.11), 15 23 24 corresponding to approximately one relapse every 20 years per patient. In comparison, other high-efficacy therapies available in the Brazilian public health system, such as natalizumab and alemtuzumab, have reported AARs of 0.15 25 and 0.05 to 0.28 26 27 respectively. Although no randomized clinical trials directly compare the efficacy of these treatments, previous observational studies 28 suggest that rituximab may be equivalent to, or even marginally superior to, natalizumab, while also offering a more favorable discontinuation profile.

Natalizumab has been the main pillar of high-efficacy treatment in Brazil. However, a major concern among patients receiving natalizumab is the risk of PML in those with positive JCV serology, often leading to treatment discontinuation after 24 months. 29 Following natalizumab withdrawal, disease activity tends to return to pretreatment levels, 30 posing a significant challenge for patients with a high inflammatory burden. A Swedish study 31 demonstrated that rituximab is superior to fingolimod after natalizumab discontinuation, showing fewer clinical relapses, fewer MRI lesions, and better tolerability. Cladribine was recently approved in the Brazilian public health system as an option for pwMS at risk of PML. However, its efficacy has been shown to be suboptimal in cases of highly-active disease compared to infusional therapies. 32 Concerning alemtuzumab, it is also considered a highly-effective option for transitioning after natalizumab; however, its adverse event profile and the requirement for monthly laboratory monitoring limit its use in certain situations. 33

The current MS treatment guidelines in the Brazilian public health system exclude primary progressive MS and do not recommend any specific therapy for progressive forms of the disease. While on-label treatments for this condition include siponimod and ocrelizumab, a meta-analysis of 13 studies 34 suggests that rituximab may also help stabilize EDSS scores. Additionally, an observational study 35 has reported that rituximab presents a similar efficacy to ocrelizumab in patients with PPMS. Rituximab remains an underused option for MS treatment in Brazil, particularly when high-efficacy therapy is needed after natalizumab or for progressive forms of the disease. Moreover, a large Swedish cohort 18 demonstrated that rituximab is more cost-effective than other DMTs, including injectables, natalizumab, and fingolimod. In Latin America, the high cost of highly-effective DMTs imposes a substantial barrier to access, disproportionately affecting patients treated outside the private healthcare sector. This economic burden contributes to significant therapeutic inequality across the region. As a lower-cost, yet highly-effective anti-CD20 therapy, rituximab has the potential to mitigate these disparities by expanding access to high-efficacy treatment within resource-constrained public health systems.

Regarding safety, the frequency of IREs in the current study was similar to previously-reported rates (7.8–25.8%). 15 23 36 These events are more common during the first infusions; they are mostly mild, and can be prevented with the use of histamine receptor antagonists and corticosteroids. Infections are the most frequently reported non-IREs in the literature, 15 23 37 which aligns with our findings. Our incidence of hypogammaglobulinemia, regarding IgG levels, was also comparable to those of previous reports (8.8–18%). 38 39 Studies 40 suggest that individuals with lower baseline IgG levels and those older than 50 years of age are at a higher risk for infections and may require immunoglobulin-replacement therapy. Additionally, higher cumulative doses of rituximab have been associated 40 with lower IgG levels during follow-up. Therefore, it is recommended that IgG, IgM, and immunoglobulin A (IgA) levels be assessed at baseline and monitored periodically throughout the rituximab treatment. 39

The main limitations of the current study arise from its observational nature, which introduces potential selection bias due to the inclusion of cases with long disease duration, higher EDSS scores compared to those of other observational studies, and a high number of prior use of DMTs. Unfortunately, our sample did not contain treatment-naïve patients, primarily due to the availability of DMTs in Brazil. WNeither did we include a comparator group, which is a goal for future work. Additionally, most of the data were collected retrospectively, and the study was limited by a small sample size. Although ARR is not the most appropriate outcome for progressive MS, SPMS and PPMS patients were included because a meaningful proportion of this group (6/20; 30%) exhibited active disease in the 2 years preceding rituximab initiation. We acknowledge that relapse-based measures present limited interpretability in progressive phenotypes, and this represents a limitation of our analysis. The small cohort also limited our ability to perform subgroup analyses and detect potential differences across patient subgroups **.** Variations in dosing intervals among patients, inconsistent MRI follow-up, and restricted access to rituximab further complicated patient selection and data consistency. Despite these challenges, the current study represents the first real-world evaluation of rituximab in Brazil, providing valuable insights into its use within the context of the country's unique public health system. Given its safety, efficacy, and potential cost-effectiveness, rituximab may offer a promising treatment option for MS in resource-limited settings such as Brazil.

The present study further reinforces the effectiveness and safety of rituximab as a treatment option for pwMS in Brazil, demonstrating significant reductions in disease activity. The study provides the first real-world dataset from the Brazilian public health system evaluating rituximab for MS, offering novel insight into its performance in a resource-limited national context. Despite challenges such as restricted access to rituximab, which led to treatment discontinuation in some cases, our findings underscore the potential of rituximab as a highly-effective and well-tolerated therapy in real-world settings. The favorable safety profile, particularly regarding the absence of severe IREs or non-IREs, adds to the growing body of evidence supporting rituximab's use in MS management, particularly in resource-limited settings such as Brazil.
